# MYCN-mediated murine cancer models

**DOI:** 10.18632/aging.101222

**Published:** 2017-04-04

**Authors:** Kristina Althoff, Alexander Schramm

**Affiliations:** Department of Medical Oncology, West German Cancer Center, University Hospital Essen, University Duisburg-Essen, Essen, Germany

**Keywords:** MYCN, neuroblastoma, neuroendocrine tumors, PanNETs, pituitary adenoma

**Figure 1:**
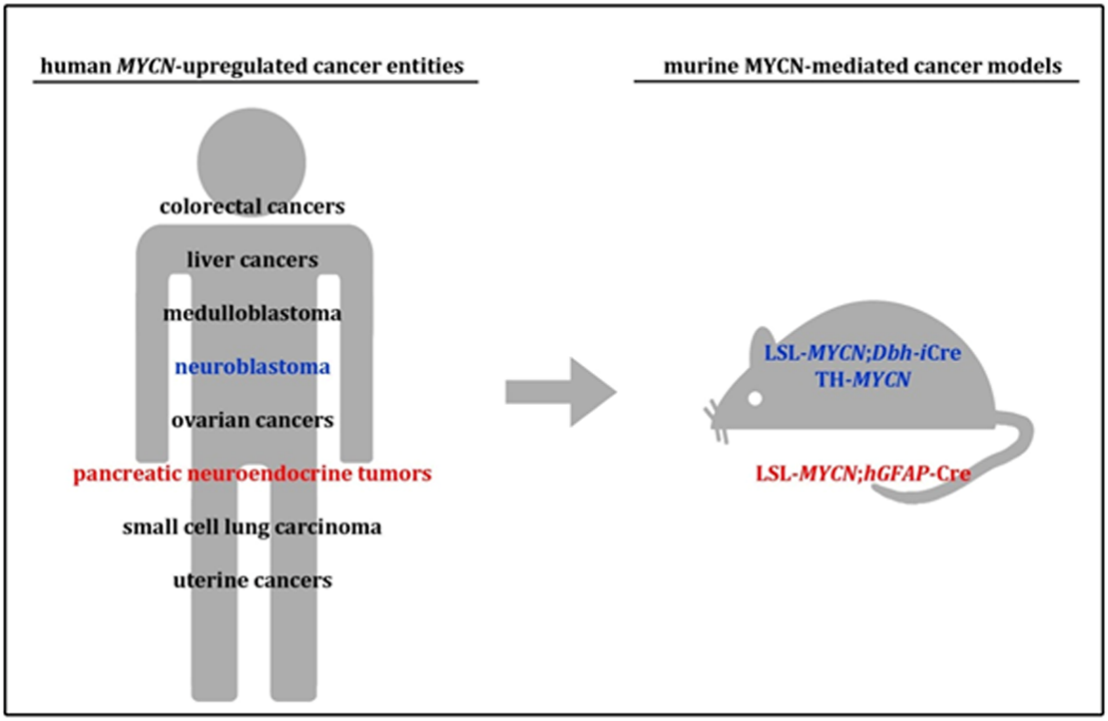
Human *MYCN*-upregulated cancer entities and appropriate established mouse models.

High expression of MYCN can facilitate tumorigenic processes in cancer entities of both, infants and adults. Elevated MYCN levels are often driven by amplification of the *MYCN*-oncogene and MYCN activity is linked to poor clinical outcome in a variety of cancer types [1]. Among solid tumors of childhood, MYCN amplification is prominently observed in neuroblastoma and medulloblastoma, whereas MYCN copy number alterations are found in several common tumors of adulthood, including small cell lung cancer (Fig. 1). Although the importance of MYCN in cancer development and maintenance of an aggressive phenotype has been widely recognized for decades, MYCN had been considered a poor drug target. With the recent advent of small molecule inhibitors indirectly interfering with MYCN functions, it now becomes essential to establish MYCN-driven in vivo tumor models that recapitulate human disease types.

For this purpose, we and others have characterized genetically engineered mouse strains that use MYCN upregulation to specifically induce tumor formation. In two murine models of MYCN-driven neuroblastoma, TH-*MYCN* [2] and LSL-*MYCN*;*Dbh-i*Cre [3], MYCN overexpression in neural crest cells resulted in tumors closely resembling human neuroblastoma in terms of tumor localization and histology, genomic aberrations and gene expression. TH-*MYCN* (Tyrosine hydroxylase-MYCN) transgenic mice have been widely used over the past two decades. Here, MYCN expression in transgenic animals is controlled by the rat TH-promoter. In LSL-*MYCN*;*Dbh-i*Cre transgenic mice, MYCN is conditionally expressed using Cre recombinase, which is active in Dbh (dopamine beta hydroxylase)-positive cells only. Of note, the latter model also is genetically well defined as LSL-*MYCN* transgene integration is confined to the *ROSA26* locus, while TH-*MYCN* transgene integration at chromosome 18q could not been fully characterized so far. Still, both model systems have their value by closely resembling human neuroblastoma in terms of histology and cytogenetic aberrations, thus confirming the oncogenic capacity of MYCN both in TH- and Dbh-positive cells.

In a recent publication, Fielitz et al. [4] described MYCN overexpression in 3 / 9 histologically validated human pancreatic neuroendocrine tumors (PanNETs). Targeting MYCN overexpression to *GFAP*-positive cells resulted in glucagon-secreting PanNETs in transgenic mice [4]. GFAP is expressed in pancreatic stellate cells, which have been described as tumor-associated, but not as tumor-initiating cells before. Overall tumor incidence of LSL-*MYCN*;*hGFAP*-Cre double-transgenic mice was 59 % after a latency between 6 and 11 months of age, when mice developed either pituitary adenomas (22 %), glucagon-producing PanNETs (62 %) or both (16 %). Interestingly, a specific MYCN signature could be derived from mRNA expression profiles of both LSL-*MYCN*;*Dbh-i*Cre neuroblastomas and MYCN-driven PanNETs, in-dicating a MYCN-specific reprogramming of the transcriptome in both tumor models. Of both tumor models, fully transformed monoclonal cell lines could be established. Grafting of these cells into immuno-compromised mice revealed that they remained addicted to MYCN function, as pharmacological MYCN inhibition using either BET-bromodomain inhibitor, JQ1, or the Aurora kinase inhibitor, MLN8237, significantly delayed tumor growth. Similar findings have been obtained in a mouse model of neuroendocrine prostate cancer using the same mouse strain (LSL-MYCN) for tissue-specific upregulation of MYCN [5]. Again, MYCN signatures were highly similar between mouse tumors and human prostate cancer transcriptome data, indicating the potential usefulness of the model to monitor treatment responses.

It can be anticipated that MYCN-driven tumor models will contribute to a better understanding of MYCN-mediated human cancer development and progression and to the identification and validation of novel therapeutic approaches. Moreover, pathway analyses are expected to reveal new insights into tumorigenesis. As MYCN overexpression has been reported for quite a number of human malignancies, the conditional LSL-*MYCN* model, combined with other specific Cre-mouse lines, will likely have the potential to reflect these diseases.
